# Human acellular dermal matrix for middle hepatic vein reconstruction in living donor liver transplantation: a prospective feasibility and safety study

**DOI:** 10.1097/JS9.0000000000005143

**Published:** 2026-04-16

**Authors:** Gwang-Sik Chun, Min-Kyung Yeo, Seok-Hwan Kim, Hyuk-Soo Eun, Sun-Jong Han, In-Sang Song

**Affiliations:** a Department of Surgery, Chungnam National University Hospital, Daejeon, South Korea; b Division of Hepatobiliary and Pancreatic Surgery, Department of Surgery, Chungnam National University Hospital, Chungnam National University College of Medicine, Daejeon, South Korea; c Department of Pathology, Chungnam National University College of Medicine, Daejeon, South Korea; d Department of Hepatology, Chungnam National University College of Medicine, Daejeon, South Korea

**Keywords:** acellular dermal matrix, graft occlusion, hepatic veins, liver transplantation, living donors, vascular, venous reconstruction

## Abstract

**Background:**

During living donor liver transplantation (LDLT), middle hepatic vein (MHV) reconstruction requires a conduit that preserves early venous outflow while avoiding late device-related complications typical of synthetic grafts. We evaluated human acellular dermal matrix (ADM) as an off-the-shelf biological conduit.

**Methods:**

In a prospective single-center cohort (2022–2025), adults undergoing LDLT with MHV reconstruction using ADM (*n* = 40) were followed. Outcomes were benchmarked against historical controls using Hemashield (*n* = 40) or polytetrafluoroethylene (PTFE; *n* = 20). Primary endpoints were early-phase patency (≤6 months) and major structural complications. Two blinded radiologists independently reviewed serial Doppler ultrasonography and contrast-enhanced computed tomography with consensus adjudication. Patency was estimated by Kaplan–Meier and compared with the log-rank test.

**Results:**

ADM achieved excellent early patency [95.0% (83.5–98.6) at 3 months; 85.0% (70.9–92.9) at 6 months]. At 18 months, patency was 62.5% for ADM versus 50.0% (PTFE) and 47.5% (Hemashield) (log-rank *P* = 0.785). No ADM graft required re-intervention, and no graft infection or duodenal invasion occurred (0/40 vs 2/60 [3.3%] in synthetic cohorts). Baseline characteristics were comparable. Case review showed that several ADM occlusions were associated with large bilomas, where secondary infection rather than intrinsic graft failure likely triggered thrombosis.

**Conclusions:**

ADM is a feasible and safe biological conduit for MHV reconstruction, delivering robust patency throughout the critical period of graft regeneration while avoiding catastrophic complications observed with synthetic materials. These hypothesis-generating results support a randomized trial and introduce the concept of ADM as a temporary biological scaffold. Given its excellent early patency and safety profile, ADM should be considered a valuable addition to the surgical armamentarium with potential to redefine success in venous reconstruction.

## Introduction

Successful venous reconstruction is a critical determinant of outcomes in living donor liver transplantation (LDLT), where unobstructed venous outflow is essential for preventing sinusoidal congestion and subsequent graft dysfunction^[^1,2^]^. While various conduits have been utilized, the ideal material remains a subject of intense investigation.

Synthetic grafts, such as expanded polytetrafluoroethylene (PTFE), have been a conventional choice due to their ready availability^[^3,4^]^. However, their use in the venous system is fraught[5] with significant limitations that create a persistent clinical challenge. The hydrophobic surface of these materials promotes protein adsorption and platelet activation, leading to a high propensity for early thrombosis. Furthermore, the profound compliance mismatch between rigid synthetic conduits and native veins creates turbulent flow, a well-established trigger for late stenosis from anastomotic intimal hyperplasia[6]. The risk of graft infection and erosion into adjacent structures, as well as devastating complications in immunosuppressed recipients, further underscores the urgent need for a superior alternative^[^7,8^]^.

To overcome these challenges, acellular dermal matrix (ADM) has emerged as a promising biological scaffold^[^9–12^]^. Processed to remove immunogenic cellular components while preserving the native extracellular matrix, ADM offers a unique theoretical advantage (Supplemental Digital Content Figure S1, available at: http://links.lww.com/JS9/H144)[13]. We hypothesized that this preserved matrix would provide a more biocompatible and thromboresistant substrate for host endothelialization, ultimately transforming into a living part of the host’s vascular system[9].

The initial months post-transplantation represent a critical window for liver regeneration, during which stable venous outflow is paramount for graft success. Therefore, a conduit that ensures high patency during this early phase could significantly improve clinical outcomes. Accordingly, the objective of this study was to rigorously evaluate the feasibility, safety, and particularly the early-phase clinical effectiveness of ADM for middle hepatic vein (MHV) reconstructions, directly comparing its performance with established synthetic materials.

This cohort study has been reported in line with the STROCSS guidelines[14].

## Materials and methods

### Study oversight and ethical conduct

This clinical study was conducted in full accordance with the principles of the Declaration of Helsinki and received approval from the authors’ Institutional Review Board (no. 2022-02-056). All participants provided written informed consent. The trial was prospectively registered at a primary registry of the WHO International Clinical Trials Registry Platform [CRIS, (blinded for review)]. The reporting of this non-randomized interventional study adheres to the Transparent Reporting of Evaluations with Non-randomized Designs (TREND) statement^15^.

### Pre-clinical evaluation in animal models

Pre-clinical animal methods are described in the Supplemental Digital Content 1, available at: http://links.lww.com/JS9/H145. The animal studies are reported in accordance with the ARRIVE guidelines (Animals in Research: Reporting *In Vivo* Experiments)[16].

### Patient selection

This non-randomized, single-center study was conducted on 100 patients who underwent MHV reconstruction during LDLT.

#### Prospective ADM cohort

Between April 2022 and January 2025, a total of 115 adult patients scheduled for LDLT were prospectively screened. After excluding 15 patients who did not meet the inclusion criteria or had other graft types, 40 patients were enrolled in the prospective ADM cohort. Inclusion criteria were (1) a graft–recipient weight ratio (GRWR) ≥0.8; (2) a model for end-stage liver disease (MELD) score ≤20; and (3) provision of written informed consent. These strict criteria were applied to minimize confounding factors related to poor recipient physiology or insufficient graft volume. Specifically, patients with high MELD scores (>20) or low GRWR (<0.8) were excluded to reduce the risks of coagulopathy-related bleeding or small-for-size syndrome (SFSS), respectively, thereby allowing for a more focused evaluation of the vascular conduit’s performance.

HIGHLIGHTS

- ADM is a feasible biological conduit for MHV reconstruction in LDLT.
- Early patency was excellent: 95% at 3 months and 85% at 6 months.
- No graft infection or duodenal invasion occurred in the ADM group.
- ADM may act as a temporary scaffold during early liver regeneration.
- Results support future randomized trials to confirm long-term outcomes.

#### Retrospective control cohort

For comparison, two retrospective control cohorts were established from patients who underwent LDLT between March 2018 and January 2025. The institutional transplant database was queried to identify patients who had received either a Hemashield (*n* = 40) or PTFE (*n* = 20) graft for MHV reconstruction.

Although the control cohorts were retrospective, key surgical and medical factors remained consistent throughout the entire study period. The surgical technique for MHV reconstruction has been standardized at the authors’ high-volume transplant center since the mid-2000s and was performed by a single surgeon with over 500 LDLT cases of experience. Furthermore, the postoperative patient management protocol did not undergo significant changes.

Control cohorts were not statistically matched (e.g., propensity score matching) due to the limited sample size. Instead, confounding was minimized by applying identical inclusion criteria to the historical pool to ensure baseline comparability. As shown in Table 1, this selection process resulted in groups with no significant differences in key variables such as recipient age, MELD score, or GRWR.

**Table 1:** Clinical profiles of patients according to graft type.

Variables	ADM group	Hemashield group	PTFE group	*P-*value
	(*n* = 40)	(*n* = 40)	(*n* = 20)	
Recipient				
Age (years)	54.6 ± 9.7	54.9 ± 6.3	52.0 ± 8.9	0.647
Sex (male, %)	23 (57.5)	29 (72.5)	12 (60.0)	0.645
Original liver disease	-	-	-	0.154
HBV-LC [*n* (%)]	11 (27.5)	22 (55.0)	6 (30.0)	0.127
Alcoholic LC [*n* (%)]	16 (40.0)	14 (35.0)	10 (50.0)	0.145
HCV [*n* (%)]	1 (2.5)	0 (0.0)	1 (5.0)	-
Others [*n* (%)]	12 (30.0)	4 (10.0)	3 (10.0)	-
MELD score	14.3 ± 7.8	13.3 ± 4.0	18.1 ± 7.4	0.099
ABO-incompatibility [*n* (%)]	16 (40.0)	11 (27.5)	3 (15.0)	0.091
GRWR	1.2 ± 0.3	1.2 ± 0.3	1.3 ± 0.4	0.239
Clavien–Dindo classification	-	-	-	-
Grade IIIa [*n* (%)]	4 (10.0)	3 (7.50)	1 (5.0)	0.075
MHV patency rate (95% CI)	-	-	-	-
At 3 months	95.0% (83.5–98.6)	85.0% (70.9–92.9)	85.0% (64.0–94.8)	0.684
At 6 months	85.0% (70.9–92.9)	60.0% (44.6–73.7)	75.0% (53.1–88.8)	0.228
At 12 months	65.0% (49.5–77.9)	50.0% (35.2–64.8)	55.0% (34.2–74.2)	0.843
At 15 months	65.0% (49.5–77.9)	50.0% (35.2–64.8)	55.0% (34.2–74.2)	0.829
At 18 months	62.5% (47.0–75.8)	47.5% (32.9–62.5)	50.0% (29.9–70.1)	0.785

ADM, acellular dermal matrix; PTFE, polytetrafluoroethylene; HBV, hepatitis B virus; LC, liver cirrhosis; MELD, model for end-stage liver disease; GRWR, graft-recipient weight ratio; MHV, middle hepatic vein; 95% CI, 95% confidence interval.

### Surgical technique: MHV reconstruction with ADM

The benchmark surgical technique involved creating a unified venous outflow for segments V and VIII. The reconstruction of MHV tributaries followed the standardized unification technique (V5 and V8)[17]. On the back table, a sheet of ADM was tailored and fashioned into a tubular conduit using a 6-0 polypropylene running suture. This ADM conduit, serving as a substitute for the MHV, was then anastomosed to the right hepatic vein (RHV) trunk on the graft, creating a single, wide common orifice for the final implantation. Specifically, this procedure followed the standardized “Single Orifice Outflow Reconstruction Technique (SORT)” described by Pamecha *et al*[18]. The ADM conduit (reconstructing V5 and V8) was conjoined with the graft RHV to create a single, unified triangular outflow orifice, which was then anastomosed to the recipient inferior vena cava (IVC).

### Postoperative management and outcome assessment

Postoperatively, a daily regimen of aspirin (100 mg) was administered for at least 1 year to patients with platelet counts above 50 000/mm^3^. The primary endpoint was graft patency, assessed by serial Doppler ultrasonography and dynamic contrast-enhanced computed tomography (CT) scans. To minimize interpretation bias, two attending radiologists, who were blinded to the graft material used, independently reviewed all imaging studies. Any discordant interpretations were resolved by joint review to reach a consensus. Inter-rater agreement for patency assessment was excellent (Cohen’s κ = 0.92). Graft occlusion was defined as the absence of detectable flow on imaging. Secondary endpoints included the incidence of postoperative complications, which were graded according to the Clavien–Dindo classification.

### Histological and statistical analysis

#### Histological analysis

Excised tissue samples from the animal models were fixed in 10% neutral buffered formalin, paraffin-embedded, and sectioned at 5 μm. Sections were stained with hematoxylin and eosin for general morphology and Masson’s trichrome for collagen deposition. For immunohistochemical analysis, primary antibodies against CD31 (endothelial cell marker) and iNOS (inflammatory marker) were used to evaluate endothelialization and the inflammatory response, respectively.

#### Statistical analysis

The sample size was determined by the number of consecutive, eligible patients who underwent the procedure during the defined study period, consistent with the exploratory nature of a feasibility study, rather than a formal power calculation. Continuous variables are presented as mean ± standard deviation and were compared using the Student’s *t*-test or Mann–Whitney *U* test. Categorical variables are presented as *n* (%) and were compared using the χ^2^ test or Fisher’s exact test. Graft patency rates were estimated using the Kaplan–Meier method, with comparisons made via the log-rank test. A two-sided *P*-value less than 0.05 was considered statistically significant. MHV patency rates are presented as % [95% confidence interval (CI)]; 95% CIs were calculated using Wilson score intervals. When Cox proportional hazard models were applied, proportional hazard assumptions were assessed using Schoenfeld residuals. All analyses were performed using SPSS version 26 (IBM Corp.).

## Results

### Pre-clinical animal findings in animal models

Pre-clinical animal findings are described in the Supplemental Digital Content 1, available at: http://links.lww.com/JS9/H145. In the rabbit patch venoplasty model, histological analysis demonstrated that the ADM was well-integrated into the native IVC wall, showing progressive endothelialization (CD31 positive) and collagen remodeling over 6 months (Supplemental Digital Content Figure S2, available at: http://links.lww.com/JS9/H144). In the porcine interposition graft model, the ADM conduits remained patent without aneurysmal changes, as confirmed by gross inspection and histology at 2 months post-implantation (Supplemental Digital Content Figure S3, available at: http://links.lww.com/JS9/H144).

### Patient demographics and baseline characteristics

**Figure 1.**
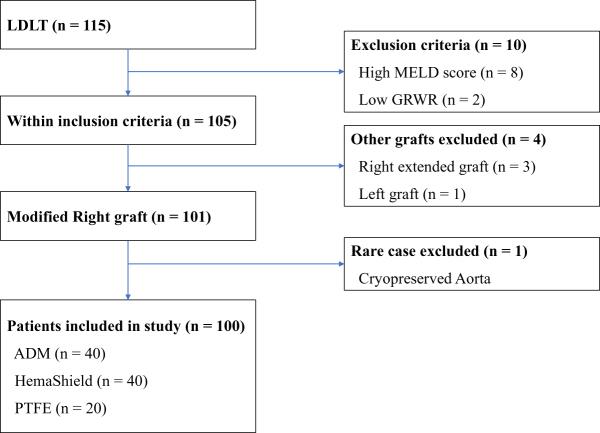
Patient selection for the comparative study. Flow diagram summarizing inclusion, exclusion, and final grouping of patients undergoing middle hepatic vein reconstruction with ADM, Hemashield, or PTFE grafts.

The patient selection process is detailed in Figure 1. A total of 100 patients requiring MHV reconstruction were included in the final comparative analysis. This included a prospective cohort of 40 patients who received an ADM graft, and two retrospective control cohorts of patients who received either a Hemashield (*n* = 40) or PTFE (*n* = 20) graft.

As summarized in Table 1, the baseline demographic and clinical characteristics were well-matched across the three groups. There were no significant differences in recipient age (*P* = 0.647), sex distribution (*P* = 0.645), underlying liver diseases (*P* = 0.154), or MELD scores (*P* = 0.099). Key surgical factors, such as the proportion of ABO-incompatible transplantations (*P* = 0.091) and the GRWR (*P* = 0.239), were also comparable among the groups.

### Postoperative complications and graft patency

#### Postoperative complications

The postoperative outcomes are summarized in Table 2. The overall rate of significant complications (Clavien–Dindo grade ≥IIIa) was not statistically different among the groups (ADM 10.0%, Hemashield 5.0%, PTFE 10.0%; *P* = 0.624). Biliary complications, such as bile leaks and strictures, were the most common events requiring intervention across all cohorts.

**Table 2:** Postoperative outcomes and complications by graft type.

Outcome	ADM (*n* = 40)	Hemashield (*n* = 40)	PTFE (*n* = 20)	*P*-value
Overall complications (Clavien–Dindo ≥ IIIa), *n* (%)	4 (10.0)	2 (5.0)	2 (10.0)	0.624
*Bile leak, n (%)*	2 (5.0)	1 (2.5)	1 (5.0)	0.799
*Biliary stricture, n* (%)	2 (5.0)	1 (2.5)	1 (5.0)	0.799
*Graft duodenal invasion, n* (%)	0 (0)	1 (2.5)	1 (5.0)	0.484
Graft-related re-intervention (stent/angioplasty), *n* (%)	0 (0)	0 (0)	0 (0)	NA*
Graft infection, *n* (%)	0 (0)	0 (0)	0 (0)	NA*

ADM, acellular dermal matrix; PTFE, polytetrafluoroethylene.

Data are presented as *n* (%). *P*-values were calculated using the χ^2^ test or Fisher’s exact test.

* NA, not applicable: statistical comparison is not possible as there were zero events in all groups.

Notably, a critical difference was observed in graft-related structural complications. Graft duodenal invasion occurred in one patient each in the Hemashield and PTFE groups, whereas no such event was observed in the ADM group (*P* = 0.484). Furthermore, no graft infections were identified in any patient. Crucially, no patient in any of the three groups required a secondary interventional procedure, such as angioplasty or stenting, for the reconstructed MHV during the follow-up period.

#### Graft patency

**Figure 2.**
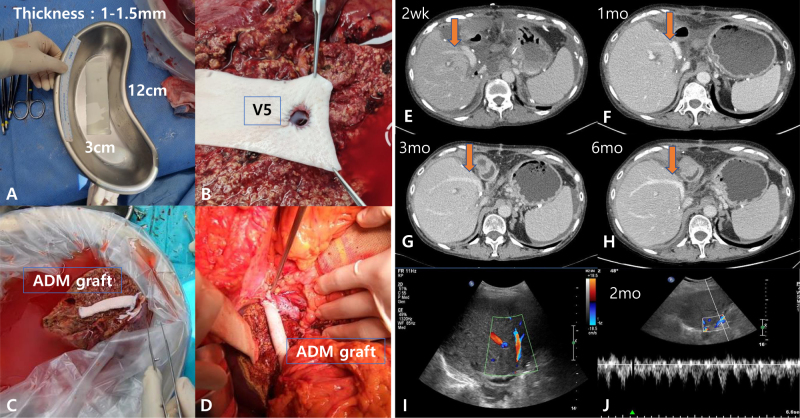
Surgical technique and postoperative imaging of ADM grafts in living donor liver transplantation. (A–D) Surgical technique for ADM conduit preparation. (A) Step 1: A sterile ADM sheet is prepared on the back table. (B) Step 2: The sheet is tailored to the measured dimensions. (C) Step 3: The sheet is fashioned into a tubular conduit using continuous sutures. (D) Step 4: The completed ADM graft is implanted for middle hepatic vein reconstruction. (E–H) Serial CT scans at 2 weeks, 1, 3, and 6 months show persistent graft patency (orange arrows). (I, J) Doppler ultrasonography at 2 months demonstrates a triphasic waveform and stable flow.

A representative case of a successful MHV reconstruction using ADM is shown in Figure 2. The surgical technique, involving the back-table preparation and implantation of the ADM conduit, is depicted in Figure 2A–D. This patient demonstrated sustained graft patency on serial contrast-enhanced CT scans up to 6 months (Fig. 2E–H) and stable, triphasic flow on Doppler ultrasonography at 2 months (Fig. 2I,J).

**Figure 3.**
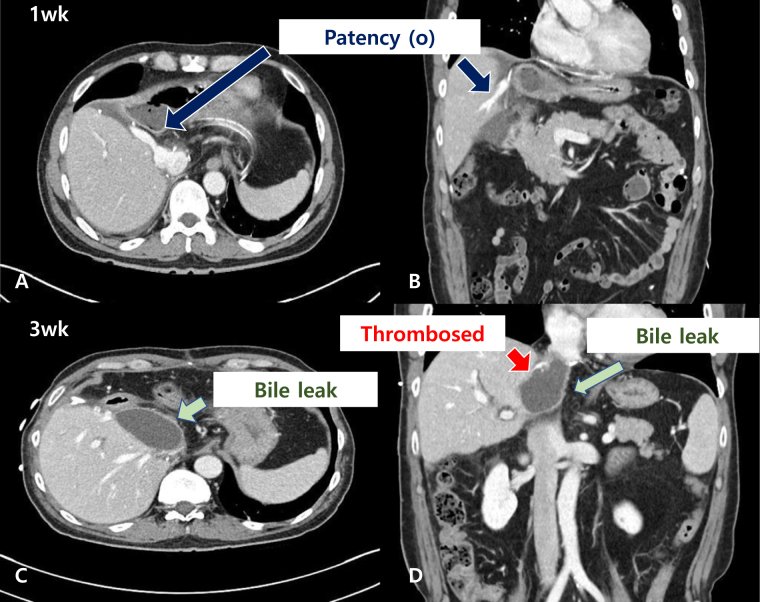
ADM graft thrombosis associated with postoperative bile leak. (A, B) CT at postoperative week 1 shows patent ADM conduit (blue arrows).(C, D) CT at postoperative week 3 demonstrates complete thrombosis (red arrows) adjacent to a large biloma (green arrows).

In the ADM cohort, graft occlusion occurred in 6 of 40 patients (15.0%) by 6 months. A detailed case review suggested that extrinsic factors, such as extensive postoperative fluid collections (bilomas), were temporally associated with graft thrombosis in at least two of these cases. One such case is detailed in Figure 3, where a patent ADM conduit at 1 week postoperatively (Fig. 3A,B) became completely thrombosed by Week 3 in the presence of a large adjacent biloma (Fig. 3C,D). Importantly, all graft occlusion events were clinically silent. None of the patients with graft thrombosis developed signs of venous congestion or SFSS, such as intractable ascites, prolonged cholestasis, or coagulopathy.

#### Comparative graft patency

The primary endpoint of graft patency was analyzed using the Kaplan–Meier method. ADM achieved excellent early patency of 95.0% [95% CI: 83.5–98.6] at 3 months and 85.0% [95% CI: 70.9–92.9] at 6 months. The estimated 18-month patency rate for the ADM grafts was 62.5% [95% CI: 47.0–75.8]. This rate was comparable to that of the PTFE (50.0% [29.9–70.1]) and Hemashield (47.5% [32.9–62.5]) grafts, with no statistically significant difference observed among the three groups (log-rank *P* = 0.785).

A subgroup analysis demonstrated that Hemashield grafts had a significantly lower long-term patency rate compared with ADM and PTFE grafts combined (log-rank *P* = 0.047). This finding underscores that Hemashield appeared to have relatively lower long-term patency for MHV reconstruction and highlights the relative stability of ADM and PTFE conduits in this setting.

## Discussion

This study provides the first prospective clinical evidence demonstrating that human ADM is a safe and feasible biological conduit for MHV reconstruction in LDLT. Beyond merely proving technical feasibility, our findings introduce a novel therapeutic paradigm: ADM functions as a “temporary biological scaffold” rather than a permanent prosthetic fixture. The primary goal of venous reconstruction in LDLT is to secure unobstructed outflow during the critical early post-transplant period, when the graft is actively regenerating and most vulnerable to congestion. Our data suggest that ensuring reliable patency during this initial regeneration window may be sufficient to achieve excellent clinical outcomes, even if the graft eventually undergoes gradual occlusion after collateral circulation has been established^[^9,11^]^. A distinct advantage of ADM over conventional synthetic options lies in its superior safety profile. Synthetic grafts, such as expanded PTFE, are permanent foreign bodies associated with a risk of catastrophic late complications, including graft infection and erosion into adjacent hollow viscera. These events are particularly devastating in immunosuppressed transplant recipients, often requiring high-risk re-interventions. In contrast, ADM is a biocompatible matrix that integrates with host tissue, thereby eliminating the risk of mechanical erosion or foreign body infection. Notably, while late occlusions occurred in the ADM group, they were clinically silent and benign, whereas synthetic graft failures often manifest as life-threatening emergencies. Thus, ADM offers a favorable risk–benefit ratio by avoiding the devastating structural complications inherent to synthetic materials^[^8,19,20^]^. Importantly, all observed ADM occlusions were clinically silent, with stable graft function maintained. Late occlusions predominantly occurred adjacent to sizable peri-graft collections and were clinically silent. While this pattern, coupled with pre-clinical evidence of early endothelialization, favors an extrinsic mechanism, definitive causality cannot be proven without human histology; thus, the contribution of intrinsic graft thrombogenicity cannot be entirely ruled out^[^21,22^]^. Taken together, these findings highlight that early effective drainage is the clinically relevant endpoint, and ADM achieves this while avoiding the devastating structural complications inherent to synthetics.

Previous studies on synthetic conduits consistently report 1-year patency rates of only 40–60%, with graft erosion or infection occurring in up to 10% of cases^[^1,23–25^]^. In contrast, our ADM cohort maintained 95% patency at 3 months and 85% at 6 months, with no catastrophic complications. Even in the six cases of ADM occlusion, most events were secondary to adjacent bilomas rather than primary graft failure, and importantly, all patients remained clinically stable^[^9,11,13^]^. These findings emphasize that ADM not only achieves superior early-phase patency but also avoids the devastating events commonly associated with synthetic grafts.

The histological findings from our rabbit and porcine models provide mechanistic explanations for the favorable clinical outcomes. The rapid endothelialization observed within weeks suggests that ADM does not remain as an inert scaffold, but actively transitions into a living, host-integrated neovessel[26]. The absence of MHC class I/II antigens confirms minimal immunogenicity, which is particularly critical in immunosuppressed transplant recipients. These mechanistic insights support the biological plausibility that ADM can sustain venous flow during the most vulnerable period of graft regeneration.

When compared with other biological substitutes, ADM occupies a unique clinical niche. Cryopreserved allogeneic veins, while effective, are limited by availability, cost, size-matching difficulties, and the risk of immunogenicity or late degeneration. While autologous peritoneum has been reported as a practical and cost-effective alternative, recent studies have noted limitations regarding its mechanical properties, including variable tissue thickness and lower tensile strength. Furthermore, peritoneal grafts carry a risk of rolling or late contracture, which may compromise long-term patency in high-risk venous reconstructions^[^27–29^]^. In contrast, ADM offers a biologically inert yet mechanically robust scaffold with standardized thickness and elasticity. This “off-the-shelf” utility eliminates the need for harvest, while its structural stability resists external compression, potentially overcoming the mechanical vulnerabilities observed with peritoneal grafts.

The successful performance of ADM in the anatomically demanding, low-flow MHV setting has broader implications. It strongly suggests potential applicability to larger vessels such as the portal vein or IVC, where higher flow may yield even more robust outcomes. Beyond transplantation, ADM may also represent a valuable option for venous replacement in pancreatic resections for borderline resectable cancers or in major abdominal trauma requiring caval or portal reconstruction, where an immediately available biological conduit can be lifesaving^[^12,30–32^]^.

## Limitations

This study has several important limitations and should be considered hypothesis-generating rather than definitive. First, the non-randomized, single-center design utilizing historical controls introduces potential selection bias; therefore, between-group analyses are primarily intended to contextualize feasibility and safety. To mitigate this risk, all procedures were performed by a single high-volume surgeon using a standardized technique, and imaging outcomes were independently evaluated in a blinded manner with excellent inter-observer agreement. Second, the sample size of the control groups was limited, particularly the PTFE cohort (*n* = 20), which restricts statistical power and increases the risk of Type II error in comparative analyses. Third, the median follow-up of 18 months provides only mid-term outcomes; longer-term data are necessary to clarify the ultimate biological fate and durability of ADM grafts. Furthermore, while imaging confirmed anatomical patency, a functional hemodynamic comparison (e.g., intravascular pressure monitoring) between graft types was not evaluated. Finally, regarding clinical implementation, although ADM carries a significantly higher upfront acquisition cost compared to synthetic grafts (PTFE or Hemashield), this expense must be weighed against the clinical costs of graft-related complications. While this study did not perform a formal health economic analysis, future studies are required to determine whether the avoidance of catastrophic synthetic graft complications—such as graft infection or organ erosion—offsets this expense and justifies the initial investment in ADM^[^33,34^]^. Additionally, as this technique relies on precise back-table preparation, technical standardization and a learning curve for graft handling must be considered for broader scalability to other centers.

Despite these limitations, the strengths of this study are notable, including prospective patient enrollment, rigorous pre-clinical validation, standardized surgical management, and blinded imaging evaluation. Together, these findings justify the execution of a definitive randomized controlled trial to establish the long-term efficacy and durability of ADM. Most importantly, they position ADM as a promising and readily available addition to the surgical armamentarium, with the potential to redefine success in venous reconstruction not by permanent patency alone, but by providing a safe and effective temporary scaffold for regeneration.

## Conclusions

In conclusion, human ADM not only serves as a feasible and safe conduit but also introduces the novel paradigm of a temporary biological scaffold in LDLT. Given its excellent early-phase patency, absence of catastrophic complications, and translational evidence from pre-clinical to clinical settings, ADM should be considered a valuable addition to the surgical armamentarium with the potential to redefine success in venous reconstruction. However, given the median follow-up of 18 months, these findings represent mid-term outcomes; longer-term durability (e.g., calcification, aneurysmal degeneration) requires prospective surveillance.

## Data Availability

The study data can be obtained from the corresponding author upon reasonable request.

## Research registration unique identifying number (UIN)

The study is registered on CRIS (The Clinical Research Information Service) https://cris.nih.go.kr/cris/search/detailSearch.do?seq=24150&search_page=L the CRIS Registration Number KCT0008243 for clinical trial.
